# Protection of Erythrocytes and Microvascular Endothelial Cells against Oxidative Damage by *Fragaria vesca* L. and *Rubus idaeus* L. Leaves Extracts—The Mechanism of Action

**DOI:** 10.3390/molecules27185865

**Published:** 2022-09-10

**Authors:** Sylwia Cyboran-Mikołajczyk, Katarzyna Męczarska, Katarzyna Solarska-Ściuk, Katarzyna Ratajczak-Wielgomas, Jan Oszmiański, Vera Jencova, Dorota Bonarska-Kujawa

**Affiliations:** 1 Department of Physics and Biophysics, Wrocław University of Environmental and Life Sciences, Norwida 25, 50-375 Wrocław, Poland; 2 Department of Histology and Embryology, Medical University of Wroclaw, Chałubińskiego 6a, 50-368 Wrocław, Poland; 3 Department of Fruit, Vegetable and Plant Nutraceutical Technology, Wrocław University of Environmental and Life Sciences, Norwida 25, 50-375 Wrocław, Poland; 4 Department of Chemistry, Faculty of Science, Humanities and Education, Technical University of Liberec, Studentska 2, 461 17 Liberec, Czech Republic

**Keywords:** HMEC-1, raspberry, wild strawberry, cytotoxicity, erythrocytes, oxidation, cells, membrane, bioactivity, *Fragaria vesca* L., *Rubus idaeus* L.

## Abstract

The aim of this work is to determine the biological activity of ellagitannins rich extracts from leaves of raspberry (*Rubus idaeus* L.) and wild strawberry (*Fragaria vesca* L.) in relation to cells and cell membranes. Detailed qualitative and quantitative analysis of phenolic compounds of the extract was made using chromatographic methods. Cytotoxic and antioxidant activities of tested extracts in relation to erythrocytes and human vascular endothelial cells (HMEC-1) were determined by using fluorimetric and spectrophotometric methods. In order to establish the influence of the extracts on the physical properties of the membrane, such as osmotic resistance and erythrocytes shapes, mobility and/or hydration of polar heads and fluidity of hydrocarbon chains of membrane lipids, microscopic and spectroscopic methods were used. The results showed that the extracts are non-toxic for erythrocytes and HMEC-1 cells (up to concentration of 50 µg/mL), but they effectively protect cells and their membranes against oxidative damage. The increase in osmotic resistance of erythrocytes, formation of echinocytes and changes only in the polar part of the membrane caused by the extracts demonstrate their location mainly in the hydrophilic part of the membrane. The results indicate that tested extracts have high biological activities and may be potentially used in delaying the ageing process of organisms and prevention of many diseases, especially those associated with oxidative stress.

## 1. Introduction

Oxidative stress in organisms leads to the development of many dangerous diseases. Its formation is the result of imbalances between processes associated with the formation of free radicals and the processes responsible for their neutralization that involve enzymatic and nonenzymatic defence mechanisms in the body [1,2]. Among the pathologies resulting from oxidative stress, the most threatening are cancerous diseases, resulting from the variety of mutations associated with oxidative damages of proteins and DNA [1,2,3,4]. Next to cancers, the second major cause of mortality of people in the world is cardiovascular diseases, such as heart attack, stroke, coronary artery disease, hypertension, etc. These diseases are closely linked to an excess of free radicals in the body as their precursors. For example, atherosclerosis, which leads to stroke or heart attack, and its domain are numerous atherosclerotic plaques, created by foam cells filled with damaged plasma lipoproteins. Interactions between the vascular wall cells, blood cells and lipoproteins in the plasma are then disturbed. At the same time, enzymes synthesised in the walls of the blood vessels catalyse many reactions that generate significant amounts of reactive oxygen species (ROS) that enhance the original atherosclerotic changes. They lead to abnormal endothelial permeability, increased retention of cell elements and lipoproteins under the endothelium and to many other adverse processes that increase dysfunction in the vascular bed [5].

Redox balance is of critical importance for the function of endothelial cells. They can be activated by various stimuli, such as thrombin or histamine, that induce a switch in their synthetic profile from basal conditions towards an activated state that is prothrombotic, pro-proliferative and vasoconstriction. These cells also play main roles in angiogenesis and vasculogenesis [6]. Dysfunction of endothelial cells is thought to be the main etiologic factor of cardiovascular diseases [7].

Free radicals can also cause damage to the morphological components of blood. Particularly, erythrocytes are continuously exposed to oxidative stress, not only because of the oxygen transport functions in the body, but also due to the high concentrations of unsaturated fatty acids in the lipid bilayer of the membrane that have, like proteins, high susceptibility to oxidation [8]. A consequence of oxidative stress is damage to the major components of the cell, causing a breach in its integrity and change in its mechanical properties, including the loss of the ability to change shape that can lead to a reduction in the mobility of erythrocytes in the blood capillaries. Free radicals, aside from peroxidation of the red blood cell membrane, cause protein aggregation and increased permeability in the membrane [9,10]. Lipid peroxidation changes the physicochemical properties of the lipid bilayer, e.g., it reduces the hydrophobicity of the lipid membrane interior, changes the organization and fluidity of the lipids and disturbs their asymmetry, causing membrane depolarization [11,12]. This leads to a decrease in membrane enzymes and transport proteins and, consequently, to the loss of membrane integrity and release of hemoglobin in the process of hemolysis.

Considering that the cause of many serious lifestyle diseases is oxidative stress, very important is the exploration of natural substances possessing high biological activity that could be used for both prevention and treatment. Secondary plant metabolites, including polyphenols, have been shown to possess a number of important properties that determine their biological activity; among others, they exhibit antioxidant, anti-inflammatory, antimutagenic, antiproliferative and the ability to modulate the physicochemical properties of cell membranes. In addition, these compounds, in contrast to traditional medicines, practically do not show side effects on biological systems, hence, the benefits arising from their use.

Leaves of raspberries *Rubus idaeus* L. (*Rubus*) and wild strawberries *Fragaria vesca* L. (*Fragaria*), as the literature data indicate, are rich and underestimated sources of substances with proven beneficial effects on the body. For centuries, they were a valuable raw material used in natural medicine for the treatment of diseases of the digestive and circulatory system. They are popularly used as decoctions for the treatment of hypertension due to their detoxifying, diuretic, stimulant and protective dermatological properties. They possess antioxidant, anticancer, gastrointestinal, antiangiogenic, anti-inflammatory, anti-aging, anti-melanogenic, radioprotector against UVB and γ-radiation-induced toxicity in human peripheral blood lymphocytes and fibroblasts, vasodilatory and anxiolytic documented activities [13,14,15,16,17,18,19,20,21,22,23,24,25,26]. These valuable properties are mainly responsible for the active compounds they contain, among which polyphenolic compounds constitute the dominant group. The main class of polyphenolics in aqueous extracts of the leaves are ellagotanins, in particular, casuarinin and its trimmer lambertianin C. These compounds are characterised by relatively large size and rather hydrophilic nature, because of the large number of hydroxyl groups they contain in the structure. This makes them effective substances against free radicals and explains their high antioxidant activity, demonstrated in the standard antioxidant tests, i.e., by the ABTS (2,2′-azino-bis(3-ethylbenzothiazoline-6 sulfonic acid) radical scavenging and ferric-reducing antioxidant power (FRAP) assay [19]. Despite the importance and the popular cultivation of *Fragaria* and *Rubus* species, studies on biological activity of their leaves in relation to the cells and biological membranes are still scarce and have so far focused on the fruit of the plants. After harvesting the fruit, the leaves of these plants are treated as a by-product; however, they are a rich source of bioactive compounds for the cosmetics and pharmaceutical industries. Therefore, the aim of this study was to determine the cytotoxicity and antioxidant activity of aqueous extracts of *Rubus* and *Fragaria* leaves in relation to selected cells in the circulatory system. Furthermore, in order to determine the molecular mechanism responsible for the activities, the basic biophysical studies that define their interaction with the membrane of erythrocytes treated as an example of the biological membrane were carried out. Exploration of the mechanism of action of these substances should expand our still lacking knowledge on their effects on biological systems and be helpful in the search for the kind of natural polyphenolic substances, dominant in the extracts from various plants, which possess optimal therapeutic/health-supporting properties, in particular, in the context of cardiovascular diseases.

## 2. Results

### 2.1. Phenolic Content

The *Rubus* and *Fragaria* leaf preparations were analysed by ultra-performance liquid chromatography-electrospray tandem mass spectrometry (UPLC–ESI-MS/MS) systems. Qualitative analysis obtained by liquid chromatography coupled to diode array detection and electrospray ionization tandem mass spectrometry (LC-DAD-MS-MS) methods and quantitative analysis obtained by UPLC-MS (quantified using DAD and MS detection) are summarized in Supplementary Materials (Tables S1 and S2; Figures S1 and S2). The comparison of the type and content (mg/g DW) of phenolic components of used extracts is summarized in Table 1.

The obtained results have shown that *Fragaria* and *Rubus* extracts are rich sources of various phenolic compounds. The main classes of the compounds they contain are ellagitannins, phenolic acids and flavonols. In the *Rubus* species, 12 phenolic compounds were identified, while 17 in the extract from the leaves of *Fragaria*. The main component in both extracts is casuarinin, which belongs to ellagitannins and its content of all identified phenolic compounds in *Rubus* and *Fragraia* extracts is approximately 83% and 64%, respectively. Additionally, *Fragaira* extract also contains other compounds of the ellagitannin group lambertianin C of high biological activity. Among the other components of both the extracts, an important group constitutes derivatives of quercetin and kaempferol and the phenolic acids: chlorogenic, neochlorogenic and gallic. Furthermore, in the leaves of *Fragaria,* apigenin-3-*O*-glucuronide was identified, belonging to flavons.

### 2.2. Toxicity of Extracts

The toxic activity of the extracts was determined in relation to erythrocytes and human vascular endothelial (HMEC-1) cells by using spectrophotometric and fluorimetric methods.

The spectrophotometric method was used to investigate the haemolytic activity of the extracts, which was determined on the basis of the concentration of haemoglobin released from unmodified and extracts modified cells. The studies showed that polyphenolic extracts from leaves of *Rubus* and *Fragaria* do not induce haemolysis after 1 and 3 h modification of the cells when used at 0.01 to 0.1 mg/mL (results not published). This means that both extracts have no toxic effect on erythrocytes.

**Figure 1:**
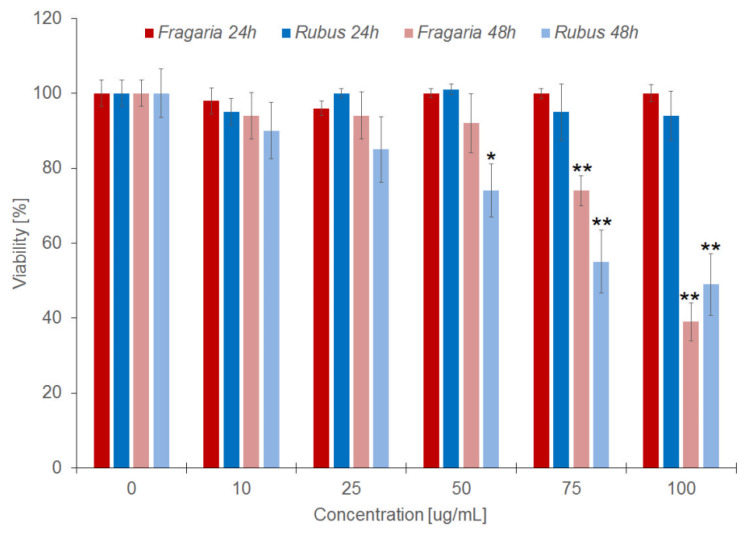
The percent of viability in cells modified by *Fragaria* and *Rubus* extracts after 24 h (grey bars) and 48 h (black bars) of incubation, calculated on the basis of MTT assay results. Statistically significant differences between modified and control cells are marked: * α = 0.05; ** α = 0.01.

The toxic activity of the extracts was also determined in relation to HMEC-1 cells by using MTT and Hoechst assays. In the first test, viability of the cells after 24 and 48 h incubation was determined on the basis of formazan concentration that arises from the reduction in MTT by oxidoreductase enzymes of living cells. The 24 h incubation of cells with the extracts applied at concentration from 10 to 100 µg/mL did not affect the number of viable cells compared to unmodified cells (Figure 1). After 48 h incubation, both the extracts cause a decrease in cell viability, particularly when used in concentrations greater than 75 and 50 µg/mL for *Fragaria* and *Rubus*, respectively (Figure 1).

**Figure 2:**
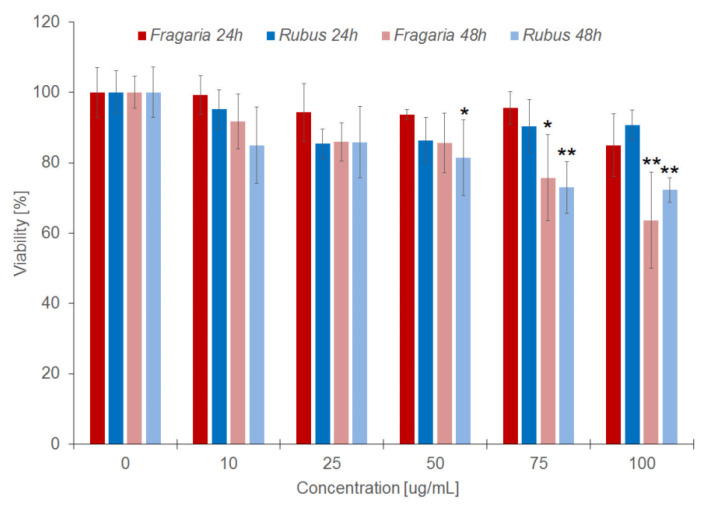
The percent of viability in cells modified by *Fragaria* and *Rubus* extracts after 24 h (grey bars) and 48 h (black bars) of incubation, calculated on the basis of the Hoechst 33342 assay results. Statistically significant differences between modified and control cells are marked: * α = 0.05; ** α = 0.01.

The results obtained in the Hoechst assay confirmed that both extracts do not affect cell viability when the incubation time is 24 h. Increasing the time to 48 h caused a decrease in cell viability, in particular at higher concentration of extracts (Figure 2). However, in this test, the decrease in cell viability was significantly less than that observed in the MTT assay.

### 2.3. Influence of Extracts on the Physical Properties of Membrane

**Figure 3:**
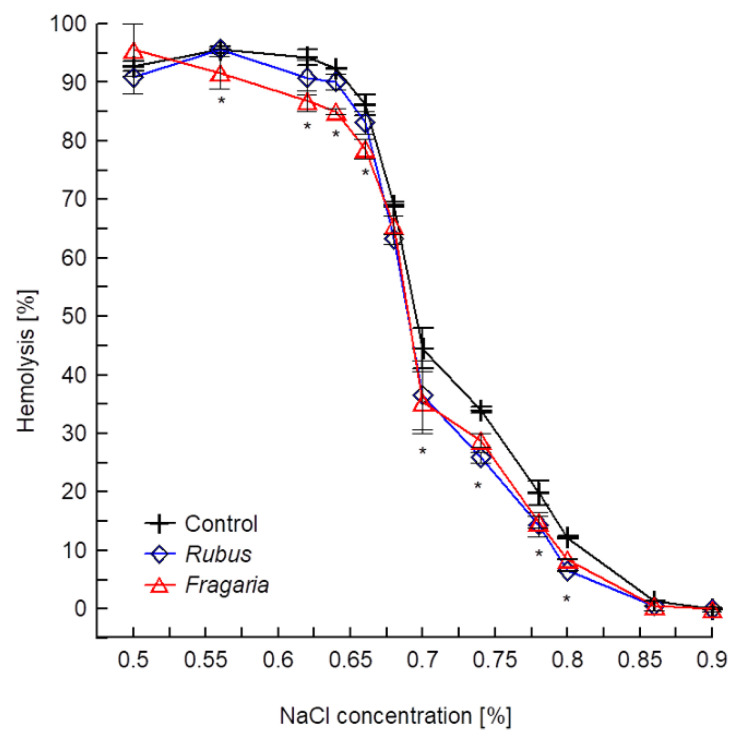
Percentage of haemolysis in *Rubus* and *Fragaria*-modified cells in hypotonic and isotonic solutions of sodium chloride. The extracts were used at 10 µg/mL concentration and the measurement was done at room temperature. Statistically significant difference between results of control and extracts modified samples are denoted (* α = 0.05).

The effect of extracts on osmotic resistance of erythrocytes was determined on the basis of haemolytic curves (percent of haemolysis vs. NaCl concentration) designed for control and modified cells. If the curve for modified cells is shifted towards lower NaCl concentrations, the red blood cells are less sensitive to changes in osmotic pressure and vice versa. The obtained results show that both extracts used at a low concentration of 10 µg/mL cause a shift in the haemolytic curves (Figure 3). It means that extract-modified cells release haemoglobin less than control cells at the same pressure difference between the external environment and the cell interior.

**Figure 4:**
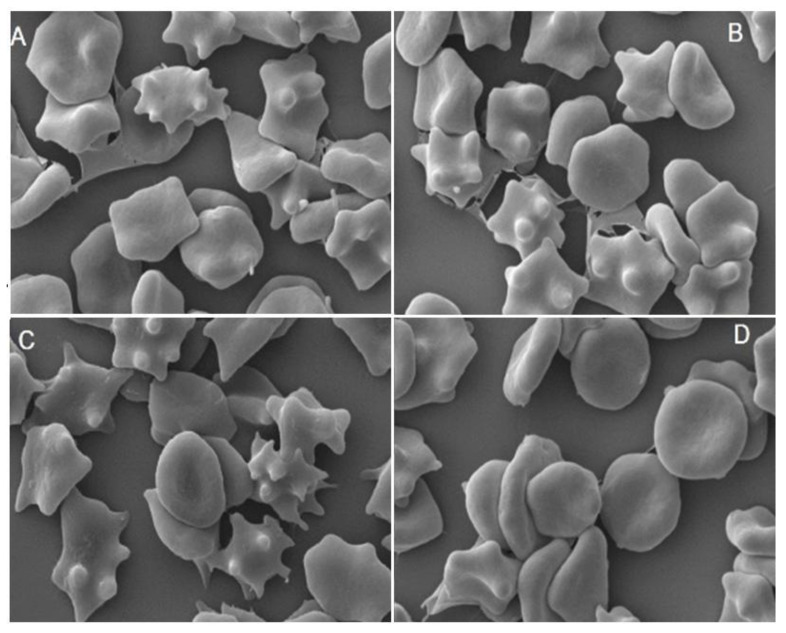
Example shapes of erythrocytes modified by *Rubus* leaf extract at 10 (**A**) and 50 µg/mL (**B**), *Fragaria* leaf extract at 50 µg/mL (**C**) and unmodified (**D**).

The demonstrated slight changes in osmotic resistance of erythrocytes indicate that extracts’ components bind to the erythrocytes membrane, making it more resistant to changes in osmotic pressure. Hence, and on the basis of images registered by electron microscope, the influence of the extracts on the shape of erythrocytes was determined. The shapes were classified according to Bessis scale [27], where various shapes are given morphological indexes, with the minus sign—echinocytes, plus—stomatocytes. The study showed that both the extracts induce changes in the shape of erythrocytes, causing formation of echinocytes (Figure 4). For a population comprising 200 cells, the percentage of the various forms of red blood cells was calculated, showing that discocytes constitute 90, 50 and 39% and echinocytes 8, 48 and 59% for control cells and those modified by *Fragaria* and *Rubus* extracts at 0.05 mg/mL, respectively. It means that *Rubus* extract is more active than *Fragaria* leaf extract, when they are used at the same concentration.

In order to determine the exact location of the compounds in the membrane of the erythrocytes, the effect of extracts on physical properties of the hydrophilic and hydrophobic part of the membrane was determined, by using two fluorescence probes that are located in those areas. Any change in the quantity and mobility of water molecules in the area of the polar heads of lipids is reflected in the value of generalized polarization (GP) of the Laurdan probe. If the GP value decreases, the membrane is more disordered and water molecules can penetrate deeper into it and vice versa. The value of anisotropy (A) in the DPH probe is related to the degree of order and mobility of the hydrocarbon chains in lipids. The decrease in A means that the membrane is more fluid and vice versa.

**Figure 5:**
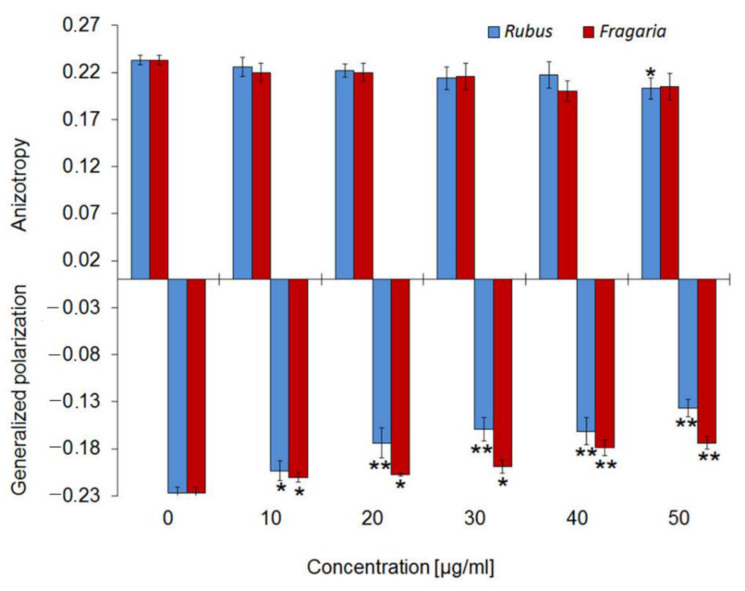
Values of generalized polarization (GP) of Laurdan probe and anisotropy (A) of DPH probe for erythrocyte membranes modified by *Fragaria* and *Rubus* leaf extracts and for unmodified cells. Statistically significant difference between results of control and extracts modified samples are denoted: * α = 0.05 and ** α = 0.01, respectively.

The studies using Laurdan probes that locate in the hydrophilic part of the membrane show that the extracts decrease generalized polarization (GP) in the probe in a concentration-dependent manner (Figure 5). Furthermore, they showed that *Rubus* extract causes significantly larger changes in the membrane area than the *Fragaria* extract. In the DPH-labelled membranes the extracts were practically not inducing changes at used concentrations. The exception is *Rubus* extract at a concentration of 50 µg/mL, which induces only a slight decrease in fluorescence anisotropy of the probe, indicating a slight increase in membrane fluidity.

### 2.4. Antioxidant Activity

The ability of extracts to inhibit 2,2′-azobis(2-amidinopropane) dihydrochloride (AAPH)-induced oxidative damage of erythrocyte membrane, erythrocytes and HMEC-1 cells was determined.

**Figure 6:**
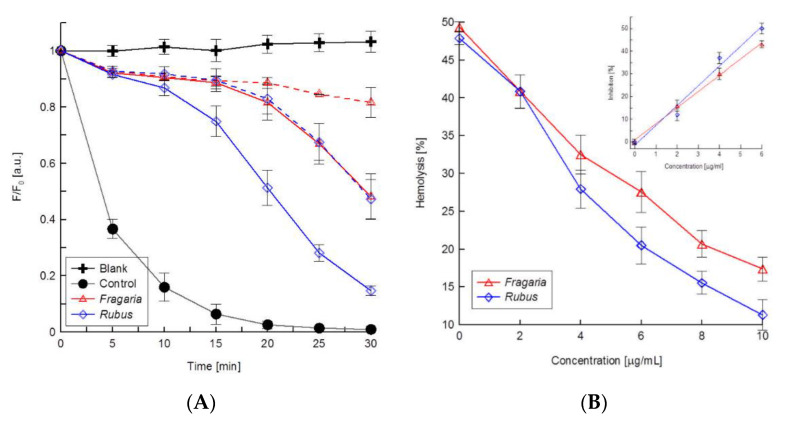
Antioxidant activity of the extracts to (**A**) erythrocyte membrane—relative fluorescence intensity of TMA-DPH probe vs. time of membrane oxidation. *Rubus* and *Fragaria* extracts were used at concentrations 4 (solid line) and 8 µg/mL (dashed line) and 3 (solid line) and 4 µg/mL (dashed lines), respectively. (**B**) erythrocytes—percentage of haemolysis of cells after 3 h oxidation by AAPH at 40 µM vs. concentration of the extracts. The dependence of the inhibition (%) of erythrocyte oxidation on the concentration of tested extracts is shown as the inserted figure.

The antioxidant activity of the extracts in relation to the erythrocyte membrane was specified using the fluorimetric method. In this method, free radicals resulting from thermal decomposition of AAPH at 37 °C oxidized TMA-DPH probe causing a decrease in their fluorescence intensity that was proportional to the degree of lipid peroxidation. Figure 6A shows the relationship between relative fluorescence intensity of TMA-DPH probe (F/F_0_) and concentration of the extracts after 30 min oxidation of erythrocyte membranes. Phenolic compounds contained in the extracts caused a concentration-dependent increase in dye fluorescence intensity. It means that used extracts inhibit lipid peroxidation in the erythrocyte membrane. Furthermore, the results have shown that *Fragaria* leaf extract much more effectively protects the erythrocyte membrane against oxidative damage, because its concentration IC_50_ (responsible for 50% inhibition of lipid peroxidation) is more than two-times lower than the IC_50_ determined for *Rubus* extract (Table 2).

Next, using a spectrophotometric method, the antioxidant activity of the extract was determined in relation to erythrocytes. During incubation of erythrocytes with the AAPH- compound, the resulting free radicals cause cellular damage, which leads to the release of haemoglobin from the cells. The concentration of released haemoglobin is proportional to absorbance measured at 540 nm and that was taken as a measure of oxidative damage to erythrocytes. With increasing concentration of extracts, a reduction in the number of haemolysed cells was observed (Figure 6B). This means that both the extracts inhibit oxidative damage of erythrocytes in a concentration-dependent manner. Additionally, the IC_50_ concentrations (Table 2) indicate that in the case of erythrocytes, the *Rubus* leaf extract provides a better protection against free-radical-induced damage.

**Figure 7:**
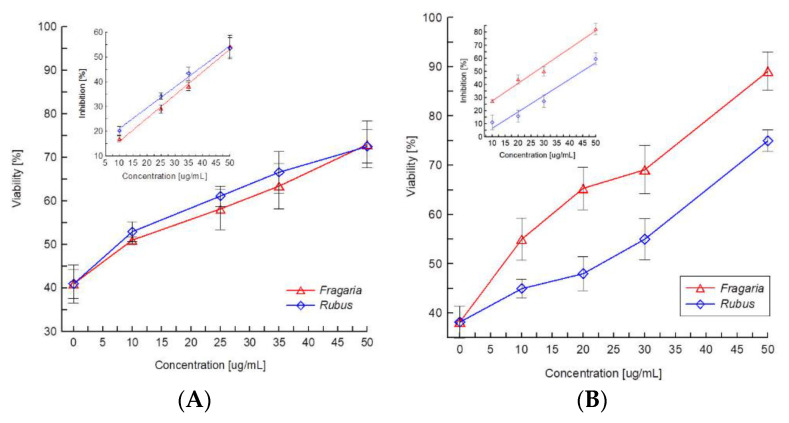
Viability (%) of HMEC-1 cells oxidized by 7.5 µM of AAPH for 24 h in the presence of different concentrations of extracts from *Rubus* and *Fragaria* leaves. (**A**) Viability was calculated on the basis of results obtained in MTT assay, (**B**) viability was calculated on the basis of results obtained in Hoechst 33342 assay. The dependences of the inhibition (%) of HMEC-1 cells oxidation on the concentration of tested extracts calculated from results of MTT (**A**) and Hoechst 33342 (**B**) assays are shown as the inserted figures.

The antioxidant activity of extracts was also determined in relation to HMEC-1 cells. To these experiments, concentrations of extracts that did not affect cells viability after 48 h modification were selected. The results show that used extracts not only protect the erythrocytes and their membrane but also vascular endothelial cells against oxidative damage induced by AAPH. In the case of the MTT assay, no difference between activities of the extracts was observed (Figure 7A). However, the results obtained in the Hoechst assay showed that *Fragaria* extract is a more effective scavenger of free radicals because the viability of oxidized cells was greater at the same concentrations of the extracts (Figure 7B).

In order to compare the antioxidant activity of the extracts with activity of substances treated as standard antioxidants, L(+) ascorbic acid was also tested. MTT and Hoechst tests showed that up to concentration of 200 µg/mL, ascorbic acid does not affect the viability of cells after 48 h of modification. Comparison of antioxidant activity of the extracts and ascorbic acid, based on IC_50_ concentrations, is presented in Table 2. The results show that both extracts are more effective in protecting erythrocyte membranes, erythrocytes and vascular endothelial cells against oxidation than ascorbic acid.

## 3. Discussion

Polyphenolic compounds endow the leaves of wild strawberry and raspberry bush with documented health-enhancing properties. Therefore, for the study, a fraction of polyphenols was extracted from the leaves of the two most popular varieties of strawberries (*Fragaria*) and raspberries (*Rubus*). Conducted chromatographic determinations have shown that in the extracts, the content of the compounds is 931 and 581 mg per gram of extract for strawberries and raspberries, respectively (Table 1). In the extract from the leaves of *Rubus,* 12 different compounds belonging to the phenolic acids, flavonols and hydrolyzing tannins were identified. The dominant component in the extract is casuarinin, which constitutes 83% of all identified polyphenolic compounds of the extract. The remaining fractions of polyphenols are flavonols, which include mainly quercetins and kaempferol, whose overall content amounts to 16.5% and phenolic acids whose contribution does not exceed 1% of all identified constituents. The content of polyphenols in wild strawberry extract was shown to be close to that in raspberry leaves, except for the derivative of apigenin that belongs to flavons. Further, in this case, the dominant group of the polyphenols is ellagitannins, among which the main component is casuarictin (63.5%) and the content of lambertannins C, which are trimers of casuarictins, is 29%. The remaining 15 identified in the extract compounds that occur in much smaller quantities, mainly neochlorogenic acid, quercetin derivatives, kaempferol and apigenin, account for 8% of the ingredients. It is also important to note that other isolated compounds, which gave significant HPLC peaks, were not clearly identified and, for that reason, they were not quantified. In general, data obtained in our study are in agreement with recently reported compositional data of *Rubus* and *Fragaria* leaves. Different tannins and flavonoids derived from quercetin and/or kaempferol were also reported in extracts of *Rubus* and *Fragaria* leaves analysed by different methods, i.e., TLC, HPLC and NMR [20,28,29,30,31,32,33].

It is believed that (poly)phenol-based nutraceuticals and functional foods might be indeed used as adjunct therapy of cardiovascular disease, but on the other hand, additional long-term randomized controlled trials, both basic and clinical, are needed to provide unequivocal evidence of their clinical usefulness [20,34]. High content of polyphenolic compounds in extracts, in particular ellagitannins, that have a high biological activity has led us to undertake a study on the influence of extracts on biological systems. The first and most important point of this type of research is exclusion or confirmation of the action itself and designation of safe permissible concentrations of test substances for cells. It is very important, because literature data indicate that polyphenols may exert toxic effects on normal and cancerous cells, depending on their type, concentration and time of action on cells [35,36]. In this connection, this study was carried out in vitro on toxicity of the extracts with regard to red blood cells and human vascular endothelial cells.

Our investigations on the haemolytic activity of the extracts show that they are not toxic to erythrocytes, as they did not cause haemolysis in a wide range of concentrations and incubation time of 3 h. The results of these tests confirmed the toxicity studies conducted with extracts using the MTT and Hoechst 33342 methods, which showed that the viability of cells treated with extracts for 24 h did not change (Figure 1), as compared with non-treated control cells. Expansion of incubation time to 48 h, by contrast, has shown that the examined extracts inhibit growth and proliferation of cells. MTT test results based on the redox activity of mitochondrial cells indicate that the tested extracts significantly reduce cellular respiration at concentrations greater than 50 and 75 µg/mL of the extract from the leaves of *Rubus* and *Fragaria*, respectively. In addition, the results obtained in the fluorimetric test, in which the survival of cells is determined on the basis of total cell’s DNA, confirmed the results obtained in the MTT test. They showed that the extracts at concentrations greater than 50 (*Rubus*) and 75 µg/mL (*Fragaria*) reduce the survival rate of vascular endothelial cells. In addition, the results indicate the lack of linear relationship between cytotoxicity of the extracts and their concentration. The results of our study confirm the reports of other authors, documenting the cytotoxic activity of ellagitannins and extracts containing them with regard to healthy and cancerous cells, e.g., keratinocyte cell line, liver, prostate, pancreatic and breast cancer cells [37,38,39].

The lack of haemolytic activity in the extracts prompted us to undertake research into their effects on the physical properties of the red blood cell. The studies of osmotic resistance of erythrocytes demonstrated that extracts slightly increase the resistance of erythrocytes to osmotic pressure (Figure 3), which points to their binding to blood cells. These results are confirmed by the photos recorded using electron microscopy, which showed that the discoidal shape of red blood cells changes to the echinocytal one under the influence of the extracts (Figure 4). The change in erythrocyte shape, according to the bilayer couple hypothesis [40], confirms that the components in the extracts bind to the membrane and indicate that they are located mainly in the outer layer of the lipid bilayer. In order to determine the exact location of the compounds in the membrane of erythrocytes, the effect of extracts on physical properties of hydrophilic and hydrophobic part of the membrane was determined, by using two fluorescence probes, Laurdan and DPH, which locate in these areas [41].

These studies have shown that compounds contained in the extracts practically do not alter fluorescence anisotropy of the DPH probe imbedded in the hydrophobic membrane area, while they cause a fall in generalized polarization of Laurdan probes anchored in the polar region of the membrane lipid heads. This means that the extract components are located not only in the outer membrane monolayer but they are associated mainly with its hydrophilic region, not penetrating to the hydrophobic interior. Their affinity to that area of the membrane arises not only from the hydrophilic nature of the components of the extracts, due to the presence of a large number of hydroxyl groups, but also from the size of ellagitannins, which are the main component of the extracts [42]. Both of these properties significantly limit the penetration of extract components to the hydrophobic inside of the red blood cell membrane. Literature data also indicate that some of polyphenols are located close to the polar-head groups of the lipids, but the location, depending on their structures and the compositions of the membrane lipids [42].

Thus, the results of this study show that ellagitannin-rich extracts from the leaves of wild strawberries and raspberries change the properties in the erythrocyte membrane by binding to it. Their location on the surface of the membrane, the lack of haemolytic and cytostatic action up to relatively high concentrations of 50 µM indicate that compounds contained in the extracts may be potentially useful for protecting living cells exposed to free radicals in the aqueous environment. Being on the surface of the membrane, they can not only capture the radicals but also constitute a protective barrier against their ingression. The ability of the extracts to protect red blood cells and their membranes and vascular endothelial cells against the harmful activity of free radicals, which arise in AAPH breakup, was examined using spectrophotometric and fluorimetric methods. These studies have shown that compounds contained in the extracts effectively protect the red blood cell membrane and erythrocytes against oxidation, the level of the protection depending on the concentration of extracts and their composition. In the case of the oxidation of erythrocyte membranes, the strawberry leaf extract provided a markedly better protection by the extract from raspberry leaves (Figure 7A). This is probably associated with the presence of a part of the extract ingredients in the medium right at the surface of the membrane, where they can effectively inhibit the free radical reactions of propagation. This is possible because research carried out with Laurdan probes showed the ingredients of this extract produce smaller changes in the polar lipid heads of the erythrocyte membrane, which points to their weaker binding in that area. With regard to the erythrocytes, the antioxidant activity in both extracts proved to be similar (Figure 7B). The results of these investigations are confirmed by the results of the determinations of the osmotic resistance and shapes of erythrocytes, which showed that the changes induced by the two extracts in physical characteristics of the red blood cell are comparable. It can, therefore, be concluded that in a similar way, the ingredients of the two extracts interact with erythrocytes, protecting them against the harmful effects of free radicals. The research on the antioxidant activity of the extracts in relation to vascular endothelial cells showed that the ingredients of both extracts protect cells against death induced by way of free radicals. The results of the MTT test indicate that both used extracts protect cells against oxidation in a similar way, as their concentrations responsible for 50% inhibition of oxidation-induced death of cells have similar values (Table 2). The measurement of reduction in MTT appears to be mainly by the mitochondrial complexes I and II, but it also may involve NADH- and NADPH-dependent energetic processes that occur outside the mitochondrial inner membrane [43]. Thus, using this method, it is possible to monitor changes in the general energetic status of the cells, but it cannot be used to separate the effect of free radicals on the individual enzymes in the mitochondrial respiratory chain [44]. The test results of the Hoechst test indicate, however, that the extract from the leaves of *Rubus* is a more effective antioxidant; the differences obtained stem from the specifics of the two tests. However, all the methods used to evaluate the antioxidant activity of the extracts unequivocally indicate that the ellagitannin-rich extracts from the leaves of the raspberries and wild strawberries are significantly more effective in inhibiting the effects of free radicals than the reference antioxidant, which is ascorbic acid (Table 2).

Ellagitannin-rich extracts from the leaves of *Fragaria* and *Rubus* do not only exhibit non-toxic effects in relation to erythrocytes and vascular endothelial cells, but effectively protect them against the harmful effects of free radicals. This is possible due to binding of the extracts’ components with a surface area of the membrane, which result in changes in their physical properties, i.e., shape, hydration or resistance to changes in osmotic pressure. Demonstrated in this work, the biological activity of extracts makes them the potential substances that may, after conducting the special tests, find use in prevention of diseases directly associated with the cardiovascular system.

## 4. Materials and Methods

### 4.1. Preparation and Phenolic Content of the Extracts

*Fragaria* and *Rubus* leaves were harvested from an experimental field of the Garden of Medicinal Plants—herbarium of the Medical University of Wroclaw, Poland. Polyphenols were isolated from leaves by extraction with water containing 200 ppm of SO_2_, the ratio of solvent to leaves being 3:1. The extracts were absorbed on Purolite AP 400 (Rochdale, UK) for further purification. The polyphenols were then eluted out with 80% ethanol, concentrated and freeze dried. By means of the above method a mixture of polyphenols was obtained using the method described earlier by Gąsiorowski et al. [45]. Phenolic compounds were identified with the HPLC/DAD method and UPLC/ESI/MS analysis described by Oszmiański et al. [46]. Phenolic content expressed as mg standard equivalent per gram of dry weight (mg/g DW).

### 4.2. Cells and Membranes

The studies were conducted on pig erythrocytes, isolated erythrocyte membranes and immortalized human microvascular endothelial cells (HMEC-1). The choice of pig erythrocytes was dictated by the fact that this cell’s percentage share of lipids is closest to that of the human erythrocyte and the blood was easily available. The erythrocytes were obtained from fresh, heparinized pig blood. For washing the erythrocytes an isotonic phosphate solution of pH 7.4 (131 mM NaCl, 1.79 mM KCl, 0.86 mM MgCl_2_, 11.79 mM Na_2_HPO_4_·2H_2_O, 1.80 mM Na_2_H_2_PO_4_·H_2_O) was used. Erythrocyte membranes were obtained from fresh blood using the Dodge method [47], whereas their content in the samples was determined on the basis of protein concentration, which was assayed using a modified Lowry method [48] and it was 100 μg/mL. The human dermal microvascular endothelial cell line HMEC-1 (ATCC^®^ CRL3243™) was purchased from American Type Culture Collection (ATCC, Manassas, VA, USA), and cultured according to its instruction.

The cells were cultured in MCDB 131 medium containing: 10% fetal bovine serum (FBS) 10 mM L-glutamine, 1 µg/mL hydrocortisone, 1% penicillin/streptomycin and 10 ng/mL epidermal growth factor (EGF) purchased in Gibco^®^ (Paisley, UK) or Merck (Darmstadt, Germany), under 5% CO_2_ in plastic flasks at 37 °C. When the cells reached 80% confluence in culture flasks, trypsin-EDTA was used to remove the cells. After trypsin neutralization by the medium, the cells were centrifuged (8 min, 700 r/min) and suspended in a small amount of medium. Next cells were counted using automated cells counter Countess™ (Invitogen™, Waltham, MA, USA) and were used in experiments or reseeded in flask.

### 4.3. Fluorescence Probes, Reagents

The fluorescent probes—1-(4-trimethylammoniumphenyl)-6-phenyl-1,3,5 -hexatriene *p*-toluenesulfonate (TMA-DPH), 6-dodecanoyl-2- dimethylaminonaphthalene (Laurdan), Hoechst 33342 nucleic acid and 1,6-diphenyl-1,3,5-hexatriene (DPH) were purchased from Molecular Probes (Eugene, OR, USA). Oxidation inducer 2,2′-azobis(2-amidinopropane) dihydrochloride (AAPH), 3-(4,5-Dimethylthiazol-2-yl)-2,5-diphenyltetrazolium bromide (MTT), Hank’s balanced salt solution (HBSS) and antioxidant standard L(+) ascorbic acid (AA) were purchased from Sigma-Aldrich, Inc. (Steinheim, Germany). All other reagents were analytically pure.

### 4.4. Toxicity of Extracts

#### 4.4.1. Haemolytic Assay

The haemolytic experiment was conducted on fresh, heparinized pig blood. Upon removing from plasma, the erythrocytes were washed four times in phosphate solution (pH 7.4) and then incubated in the same solution but containing appropriate amounts of the compounds studied. The modification was conducted at 37 °C for 1 h or 3 h, each 1 mL sample containing erythrocyte suspension of 1.2% haematocrit and 1–100 µL of extracts (10 mg/mL in phosphate buffer), stirred continuously. After modification, 2 mL of phosphate buffer was added and samples were centrifuged and the supernatant assayed for haemoglobin content using the spectrophotometric method described earlier in Cyboran-Mikołajczyk et al. [49] with minor modification. Haemoglobin concentration in the supernatant measured (Spekord 40, AnalitykJena, Jena, Germany) at 540 nm wavelength, expressed as percentage of haemoglobin concentration in the supernatant of totally haemolyzed cells, was assumed as the measure of the extent of haemolysis.

#### 4.4.2. Viability Assays

The extract effect on the viabilities of HMEC-1 cells was assessed by MTT (tetrazolium dye 3-(4,5-dimethylthiazol-2-yl)-2,5-diphenyltetrazolium bromide) and Hoechst 33342 assays described earlier [49]. The cells (5 × 10^3^/well) were seeded in 96-well flat-bottom culture plates with 100 μL culture medium and incubated for 24 h. Subsequently, medium was removed and 200 μL culture media with different concentrations (10–100 μg/mL) of extracts, dissolved in medium, was added into the wells. Then the plates were incubated at 37 °C under 5% CO_2_ for 24 or 48 h. Medium was added to the control wells. Medium was added to the control wells. After incubation, in MTT assay, medium was removed by aspiration and to cells 50 µL of MTT (0.5 mg/mL) was added. Cells were incubated in the dark for 2 h (37 °C, 5% CO_2_). After incubation, the medium was removed and formazan crystals formed were dissolved in dimethyl sulfoxide (DMSO) and absorbance of the plate was read at 570 nm using EL311 Microplate Reader (Behring, King of Prussia, PE, USA). Cell viability was calculated as the ratio of the absorbance measured for the extract-modified cells and unmodified and expressed as a percentage.

In Hoechst test, after cell incubation with extracts, medium was removed and cells were washed twice with HBSS buffer containing 1% of bovine serum albumin and frozen at −70 °C. Next, the cells were thawed at room temperature and refrozen after addition of 100 mL distilled water added to them. After throwing the plates at room temperature, 100 µL of HVAB solution (0.5% Hoechst in TNE solution containing 2 M NaCl, 1 mM EDTA, 10 mM tris-HCl, pH 7.4) was added to each well. Next, cells were incubated for 15 min in the dark at 37 °C, mixed and the fluorescence intensity of the probe was measured at 355 and 460 nm for excitation and emission, respectively (Fluoroskan Ascent FL, Thermo Scientific, Waltham, MA, USA). Cell viability was calculated as the ratio of the fluorescence intensity of the Hoechst 33342 probe for extracts modified to unmodified cells and expressed as a percentage.

### 4.5. Influence of Extracts on the Physical Properties of Membrane

#### 4.5.1. Osmotic Resistance

Osmotic resistance experiments were performed on fresh pig blood and investigated using the spectrophotometric method described earlier in Cyboran-Mikołajczyk et al. [49] with minor modification. After removing the plasma and leukocytes, the erythrocytes obtained were washed 310 mOsm PBS isotonic solution. Next, 1.2% red cells suspension containing extracts of 0.01 mg/mL or 0.05 mg/mL concentration was prepared and left for 1 h at 37 °C. After this modification, the suspension of erythrocytes was centrifuged. From the cell sediment was taken 100 µL samples of extract—modified cells and suspended in test tubes containing (0.5–0.86%) NaCl solutions and to an isotonic (0.9%) NaCl solution. Percentage of haemolysis was measured with a spectrophotometer at 540 nm wavelength.

#### 4.5.2. Shape of Erythrocytes

The effect of extracts on the shape of erythrocytes was investigated using an electron microscope. The microscopic method used was previously described in Cyboran-Mikołajczyk et al. [49] with a slight modification. The red blood cells were modified with *Rubus* and *Fragraia* leaf extracts at a concentration of 0.05 mg/mL. The material ultrastructure was analysed using a scanning microscope (EVO LS15 ZEISS) with SE1 detector, under high-vacuum and accelerating voltage EHT = 20 kV.

Various forms of erythrocyte cells have been assigned morphological indicators according to the Bessis scale [27], which for cup shapes—stomatocytes have negative values from −1 to −4 and for crenated shapes—echinocytes—positive values from 1 to 4.

#### 4.5.3. Fluidity and Mobility/Hydration of the Polar Head of the Membrane Lipids

The effect of polyphenols on the fluidity and mobility/hydration of lipids in the erythrocyte membrane (ghosts) was investigated using the fluorimetric method. Fluorescence intensity was measured by using fluorescent probes: Laurdan and DPH. These fluorescent probes were used because each of them is incorporated into different regions of the lipid bilayer. The active part (fluorophore) of the DPH probe is located in the hydrophobic and that of Laurdan in hydrophilic regions of the bilayer, respectively. Such differentiated incorporation of the probes gives an insight into the structural changes caused by incorporation of *Rubus* and *Fragaria* leaf extracts [41]. The erythrocytes membranes were suspended in an isotonic phosphate solution of pH 7.4, at a quantity such that the protein concentration in the samples amounted to approx. 100 µg/mL. The control samples contained erythrocyte ghosts or liposome suspension and a fluorescent probe, while the investigated samples contained in addition appropriate concentrations of the compound studied. Fluorescence intensity was measured by using fluorescent probes, whose concentration in the samples was 10 µM, while concentrations of the extracts were within the range 0.005–0.05 mg/mL at a temperature of 37 °C. The measurements were conducted with a fluorimeter (CARRY Eclipse of VARIAN) equipped with a Peltier temperature controller DBS (temp. accuracy ±0.1 °C). The excitation and emission wavelengths for probe DPH probe were λ_ex_ = 360 nm, λ_em_ = 425 nm. The excitation wavelength for Laurdan was 360 nm and the emitted fluorescence was recorded at two wavelengths, 440 and 490 nm.

On the basis of the measured fluorescence intensity of probes, the values of fluorescence anisotropy (A) for DPH probe and generalized polarization (GP) for the Laurdan probe was calculated using the formula described by Lakowicz [41].

### 4.6. Antioxidant Activity

#### 4.6.1. Erythrocyte Membranes

The antioxidant activity of extracts towards biological membranes was determined by fluorimetric methods using AAPH as oxidation inducer. A TMA-DPH fluorimetric probe was used in this experiment. Erythrocyte ghosts with and without (control) additions of extracts were suspended in a phosphate buffer of pH 7.4 and treated with the chemical oxidation inducer AAPH for 30 min. Free radicals, released in the process of membrane lipid oxidation, cause quenching of TMA-DPH fluorescence, decreasing the fluorescence intensity. A Cary Eclipse (Varian) spectrofluorimeter was used to measure free-radical concentrations in the samples. Excitation and emission wavelengths were λ_ex_ = 364 nm and λ_em_ = 430 nm. The fluorimetric method, leading to the determination of the percentage inhibition of oxidation by the tested extracts, was previously described in [49].

#### 4.6.2. Erythrocytes

To test the effect of extract on haemolysis induced by free radicals, RBCs were pre-incubated with varying concentrations of extracts at 37 °C for 1 h. Haemolysis of RBCs was carried out by mixing a 2% suspension of RBCs (unmodified or modified by extracts) in phosphate buffer of pH 7.4 with AAPH solution (final concentration 40 mM). This reaction mixture was incubated for 2 h at 37 °C. After incubation, samples were centrifuged for 15 min (2000× *g*) at 23 °C. The extent of haemolysis was determined spectrophotometrically by measuring the absorbance of supernatant at 540 nm. For reference, RBCs were treated with redistilled water and the absorbance of the hemolysate was used as 100% haemolysis. The IC_50_ concentrations of extracts, responsible for 50% inhibition of haemolysis induced by AAPH, were determined and compared with IC_50_ values determined for the standard antioxidant L(+) ascorbic acid.

#### 4.6.3. HMEC-1 Cells

Antioxidant activity of extracts in relation to HMEC-1 cells was determined using MTT and Hoechst 33342 assays with minor modification. The HMEC-1 cells (5 × 10^3^/well) were seeded in 96-well flat-bottom culture plates with 100 μL culture medium and incubated for 24 h. Subsequently, the medium was removed and 100 μL media containing various concentrations (10–200 μg/mL) of extracts was added into the wells (100 µL medium to the control). After 24 h incubation of cells with extracts, 100 µL of 15 µM AAPH was added (final concentration in well was 7.5 µM). The time of oxidation of the cells by AAPH was 24 h. After incubation, the procedures were exactly the same as described above (viability assays paragraph). The antioxidant activity of the extracts was determined on the basis of extracts’ ability to inhibit oxidative damage of the cells. The percentage of inhibition was calculated from the formula:

(1) $$\%\;of\;oxidation\;inhibition\;=\;\frac{\left(V_{x}-V_{k}\right)}{\left(100_{}-V_{k}\right)}\cdot 100\;\%$$

where *V_x_* = viability of cells oxidized by AAPH for 24 h in the presence of the compounds, *V_k_* = viability of cells oxidized by AAPH for 24 h without the compounds.

### 4.7. Statistical Analysis

Statistical analysis of the results was performed using the STATISTICA 12.0 (StatSoft PL) software. Statistical analysis was conducted using the Dunnett test (post hoc test—ANOVA) at significance level α = 0.01 or α = 0.05. All the experiments were conducted in at least three replicates, the results being presented as mean ± standard deviation.

## 5. Conclusions

In this study, the biological activity of *Fragaria vesca* L. and *Rubus idaeus* L. leaf extracts against cells and cell membranes was determined. Detailed qualitative and quantitative analysis of phenolic compounds contained in the extracts indicated ellagitannins as the dominant components in the extracts. The results of the cytotoxic and antioxidant activity of *Fragaria* and *Rubus* in relation to erythrocytes and human vascular endothelial cells (HMEC-1) showed that the extracts effectively protect these cells and their membranes against oxidative damage and show no toxicity towards them. The study of changes in the physical properties of the erythrocyte membrane in the presence of extracts showed an increase in osmotic resistance, the formation of echinocytes and changes only in the polar part of the membrane, which proves their location mainly in the hydrophilic part of the membrane.

The results of the research indicate that the extracts from *Fragaria* and *Rubus* leaves, rich in ellagitannins, not only show a non-toxic effect on erythrocytes, but also effectively protect them against the harmful effects of free radicals. This is possible due to binding of the extracts’ components with a surface area of the membrane, which result in changes in their physical properties, i.e., shape, hydration or resistance to changes in osmotic pressure. Demonstrated in this work, the biological activity of extracts makes them the potential substances that may, after conducting the special tests, find use in prevention of diseases directly associated with the cardiovascular system and in the prevention of many other diseases, especially those related to oxidative stress.

## Figures and Tables

**Table 1:** The contents (mg/g DW) of phenolic compounds of the *Rubus idaeus* L. (*Rubus*) and *Fragaria vesca* L. (*Fragaria*) extracts determined on the basis of UPLC-ESI-MS/MS method.

Compounds	*Rubus* [mg/g]	*Fragaria* [mg/g]
Neochlorogenic acid	0	5.05
Chlorogenic acid	1.22	0
Ellagic acid	2.21	3
*p*-Coumaroylquinc acid	0.41	0
Ellagitannins Lambertianin C	0	267.98
Ellagitannins hex (casuarinin)	483.33	592.55
Quercetin-3-*O*-rutinoside	0	8.78
Quercetin-3-*O*-glucoside- glucuronide	3.44	0
Quercetin-3-*O*-rutinoside	26.62	2.25
Quercetin-3-*O*-galactoside	0	2.2
Quercetin -3-*O*-glucuronide	5.37	15.12
Quercetin-3-*O*-glucoside	0	6.6
Kaempferol-3-*O*-rutinoside	37.92	9.19
Kaempferol -3-*O*-glucoside- glucuronide	2.32	0
Kaempferol-3-*O*-rhamnoside-7-*O*-galacturonide	18.03	0
Luteolino-3-*O*-glucoronide	0	2.74
Kaempferol-3-*O*-glucoside	0	1.68
Kaempferol-3-*O*-glucoside-rhamnoside-7-*O*-rhamnoside	0	1.34
Kaempferol-3-*O*-glucoside-7-*O*-rhamnoside	0	1.47
Kaempferol-3-*O*-glucuronide	0.27	2.24
Quercetin-3-*O*-6-acetylglucoside	0	1.36
Apigenin-3-*O*-glucoronide	0	7.88
Isorhamnetin-3-*O*-rhamnoside	0.74	0
Total	**581.88**	**931.43**

**Table 2:** IC_50_ concentrations of *Rubus* and *Fragaria* leaf extracts that inhibit oxidative damage of erythrocyte membrane lipids (RBC membrane), erythrocytes (RBC) and human vascular endothelial cells (HMEC-1).

Object	Test	*Fragaria* [µg/mL]	*Rubus* [µg/mL]	Ascorbic Acid [µg/mL]
RBC membrane	fluorimetric	3.15 ± 0.16	7.30 ± 0.51	20.5 ± 1.78
RBC	spectrophotometric	6.05 ± 0.61	5.63 ± 0.68	32.57 ± 3.16
HMEC-1	MTT	46.0 ± 6.6	45.4 ± 5.8	>100
	Hoechst 33342	26.9 ± 2.7	45.5 ± 3.5	>100

## Data Availability

The data used to support the findings of this study are available from the corresponding authors.

## Supplementary Materials

The following are available online at https://www.mdpi.com/article/10.3390/molecules27185865/s1, Table S1. The content [mg/g] and characterization of phenolic compounds of the *Rubus* L. leaf preparation of using their spectral characteristic in UPLC-DAD (retention time, λ_max_) and negative ions in UPLC–ESI-MS; Table S2. The content [mg/g] and characterization of phenolic compounds of the *Fragaria vesca* L. preparation of using their spectral characteristic in UPLC-DAD (retention time, λ_max_) and negative ions in UPLC–ESI-MS; Figure S1. UPLC-UV max plot chromatograms of phenolic compounds of the *Rubus* L. leaf preparation; Figure S2. UPLC-UV 360 nm chromatogram of phenolic compounds of the *Fragaria vesca* L. leaf preparation.

## Sample Availability

Samples of the compounds are not available from the authors.
