# Supplementary material for: Protection of Erythrocytes and Microvascular Endothelial Cells against Oxidative Damage by Fragaria vesca L. and Rubus idaeus L. Leaves Extracts—The Mechanism of Action
Source: Molecules. 2022 Sep 10;27(18):5865. doi: 10.3390/molecules27185865 (PMC9501125; doi:10.3390/molecules27185865)
Supplement: Supplementary file 1 [file molecules-27-05865-s001.zip › molecules-1904120-supplementary.pdf]

**Protection of Erythrocytes and Microvascular Endothelial Cells against Oxidative Damage by Fragaria vesca L. and Rubus idaeus L. Leaves Extracts — The Mechanism of Action**

Sylwia Cyboran-Mikołajczyk, Katarzyna Męczarska, Katarzyna Solarska-Ściuk, Katarzyna Ratajczak-Wielgomas, Jan Oszmiański, Vera Jencova and Dorota Bonarska-Kujawa

**Supplementary Materials**

**Table S1.** The content [mg/g] and characterization of phenolic compounds of the *Rubus L.* leaf preparation of using their spectral characteristic in UPLC-DAD (retention time, $\lambda_{max}$) and negative ions in UPLC–ESI-MS.

| Compounds | Content [mg/g] | $R_t$ (min) | $\lambda_{max}$ (nm) | [MS-] | [MS-MS-] |
|---|---|---|---|---|---|
| 1. Neochlorogenic acid | 5.05 | 2.34 | 323 | 353.0866 | 235.9249/191.0553/146.9378 |
| 2. Ellagitannins Lambertianin C | 267.98 | 5.12 | 240 | 1401.3730 | 633.075 |
| 3. Ellagitannins hex (casuarinin) | 592.55 | 5.59 | 240 | 935.0760 | 633.075/ 300.9999 |
| 4. Quercetin-3-O-rutinoside | 8.78 | 6.37 | 352 | 609.1080 | 301.0350 |
| 5. Ellagic acid | 3.00 | 6.60 | 364 | 301.000 | |
| 6. Quercetin-3-O-rutinoside | 2.25 | 6.98 | 354 | 609.1427 | 463.0397/301.0277/151.0034 |
| 7. Quercetin-3-O-galactoside | 2.20 | 7.14 | 353 | 463.0843 | 301.0277/151.0034 |
| 8. Quercetin -3-O-glucuronide | 15.12 | 7.27 | 351 | 477.0670 | 301.0277/151.0034 |
| 9. Quercetin-3-O-glucoside | 6.60 | 7.37 | 352 | 463.0843 | 301.0277/151.0034 |
| 10. Kaempferol-3-O-rutinoside | 9.19 | 7.48 | 350 | 593.1559 | 447.0968/285.0187 |
| 11. Luteolino-3-O-glucoronide | 2.74 | 7.54 | 340 | 461.0710 | 285.0187 |
| 12. Kaempferol-3-O-glucoside | 1.68 | 8.05 | 346 | 447.0916 | 285.0313/229.0230 |
| 13. Kaempferol-3-O-glucoside-rhamnoside-7-O-rhamnoside | 1.34 | 8.28 | 346 | 739.1930 | 593.1559 |
| 14. Kaempferol-3-O-glucoside-7-O-rhamnoside | 1.47 | 8.38 | 346 | 593.1559 | 431.0968/285.0187 |
| 15. Kaempferol -3-O-glucuronide | 2.24 | 8.51 | 346 | 461.0710 | 285.0187 |
| 16. Quercetin-3-O-6-acetylglucoside | 1.36 | 8.85 | 350 | 505.0980 | 447.0397/301.0277/151.0034 |
| 17. Apigenin-3-O-glucoronide | 7.88 | 8.95 | 338 | 445.0710 | 269.0450 |
| **Total** | **931.43** | | | | |

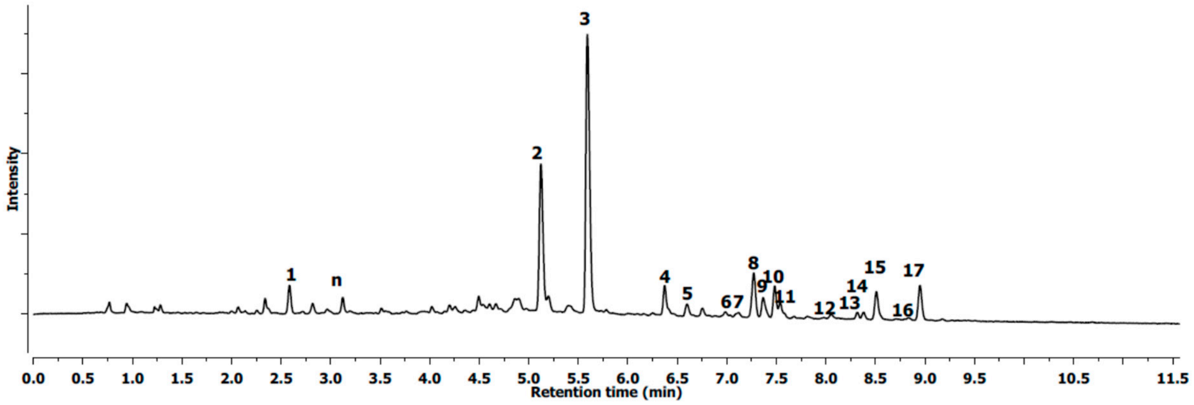


**Figure S1.** UPLC-UV max plot chromatograms of phenolic compounds of the *Rubus L.* leaf preparation (refer to Table 1 for the identification of time retention peaks).

**Table S2.** The content [mg/g] and characterization of phenolic compounds of the *Fragaria vesca L.* preparation of using their spectral characteristic in UPLC-DAD (retention time, $\lambda_{max}$) and negative ions in UPLC–ESI-MS.

| **Compounds** | **Content [mg/g]** | **$R_t$ (min)** | **$\lambda_{max}$ (nm)** | **[MS-]** | **[MS-MS-]** |
|---|---|---|---|---|---|
| 1.Chlorogenic acid | 1.22 | 4.69 | 323 | 353.0866 | 235.9249/191.0553/146.9378 |
| 2. Quercetin-3-O-glucoside- glucuronide | 3.44 | 5.69 | 352 | 639.1190 | |
| 3.p-Coumaroylquinc acid | 0.41 | 6.05 | 312 | 337.0560 | |
| 4. Quercetin-3-O-rutinoside | 26.62 | 6.32 | 352 | 609.1080 | 447.0916/301.0277/151.0034 |
| 5.Ellagic acid | 2.21 | 6.54 | 364 | 301.000 | |
| 6.Kaempferol-3-O-glucoside-glucuronide | 2.32 | 6.60 | 354 | 623.123 | 447.0968/285.0313/229.0230 |
| 7. Ellagitannins hex (casuarinin) | 483.33 | 6.85 | 245 | 934.0707 | 783.069/633.075/ 300.9999 |
| 8. Quercetin -3-O-glucuronide | 5.37 | 7.21 | 351 | 477.0670 | 301.0277/151.0034 |
| 9.Kaempferol 3-O-rhamnoside-7-O-galacturonide | 18.03 | 7.30 | 345 | 607,1290 | 285.0313 |
| 10.Kaempferol-3-O-rutinoside | 37.92 | 7.42 | 350 | 593.1559 | 447.0968/285.0187 |
| 11.Kaempferol -3-O-glucuronide | 0.27 | 8.44 | 346 | 461.0710 | 285.0187 |
| 12.Isorhamnetin-3-O-rhamnoside | 0.74 | 8.63 | 370 | 461.0720 | 315.0140 |
| **Total** | **581.88** | | | | |

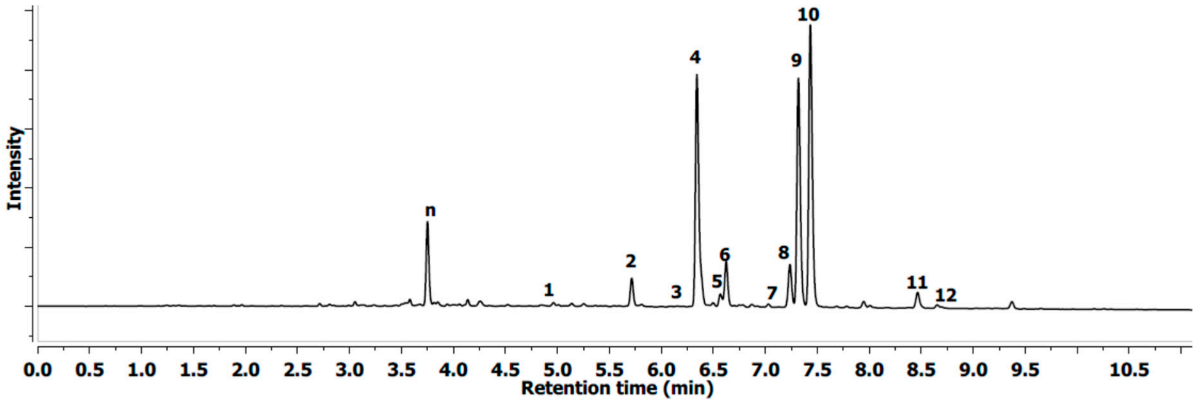


**Figure S2.** UPLC-UV 360 nm chromatogram of phenolic compounds of the *Fragaria vesca L.* leaf preparation (refer to Table 1 for the identification of time retention peaks).
